# Explaining ecological clusters of maternal depression in South Western Sydney

**DOI:** 10.1186/1471-2393-14-47

**Published:** 2014-01-24

**Authors:** John Eastwood, Lynn Kemp, Bin Jalaludin

**Affiliations:** 1 School of Public Health and Community Medicine, University of New South Wales, Sydney, NSW 2052, Australia; 2 Centre for Health Equity Training Research and Evaluation, University of New South Wales, Sydney, NSW 2052, Australia; 3 Department of Community Paediatrics, South Western Sydney Local Health District, Locked Bag 7279, Liverpool BC, NSW 1871, Australia; 4 School of Women's and Children's Health, University of New South Wales, Sydney, NSW 2052, Australia; 5 School of Public Health, University of Sydney, Sydney, NSW 2006, Australia

**Keywords:** Postnatal depression, Social capital, Poverty, Access, Ethnic segregation, Qualitative, Realism, Business social responsibility

## Abstract

**Background:**

The aim of the qualitative study reported here was to: 1) explain the observed clustering of postnatal depressive symptoms in South Western Sydney; and 2) identify group-level mechanisms that would add to our understanding of the social determinants of maternal depression.

**Methods:**

Critical realism provided the methodological underpinning for the study. The setting was four local government areas in South Western Sydney, Australia. Child and Family practitioners and mothers in naturally occurring mothers groups were interviewed.

**Results:**

Using an open coding approach to maximise emergence of patterns and relationships we have identified seven theoretical concepts that might explain the observed spatial clustering of maternal depression. The theoretical concepts identified were: Community-level social networks; Social Capital and Social Cohesion; "Depressed community"; Access to services at the group level; Ethnic segregation and diversity; Supportive social policy; and Big business.

**Conclusions:**

We postulate that these regional structural, economic, social and cultural mechanisms partially explain the pattern of maternal depression observed in families and communities within South Western Sydney. We further observe that powerful global economic and political forces are having an impact on the local situation. The challenge for policy and practice is to support mothers and their families within this adverse regional and global-economic context.

## Background

We take as our starting point the proposition that maternal stress and depression adversely impact on the developmental origins of health and disease. We cannot yet be certain of the biological level mechanisms that alter the genotypic and phenotypic response to perinatal adversity but the triggering of genetic, neuroendocrine and physiological mechanisms by psychological and nutritional stress are regarded as strong contenders [1-3]. Postnatal depression and anxiety have also consistently been demonstrated to adversely impact on maternal-infant interaction and attachment [4-6] and subsequent child cognitive, language, behavioural and psychological problems [7-15].

The majority of estimates of postnatal depression prevalence in industrialised countries are between 13-20 percent of women [16,17]. A recent large Australian study found 15.5 percent of postnatal women had depressive symptoms [18] and in our South Western Sydney study (n = 25,455), using the Edinburgh Postnatal Depression Scale (EPDS), we found that 12 percent and 6.2 percent of women had EPDS scores of >9 and >12 respectively [19].

That depression is caused by psychological stress is increasingly certain [20,21]. However, stress in itself may not be a sufficient mechanism and the tendency of stress to cause depression is almost certainly conditional on personal characteristics and social and cultural context. There is a large body of empirical research related to predictors of postnatal depression. Beck undertook a meta-analysis of 84 published studies of postnatal depression and identified 13 significant predictors. They were: pre-natal depression, self-esteem, childcare stress, prenatal anxiety, life stress, social support, marital relationship, history of previous depression, infant temperament, maternity blues, marital status, socioeconomic status, and unplanned/unwanted pregnancy [22].

In our individual-level South Western Sydney studies of postnatal depression, predictors have included: financial stress, infant behaviour, emotional support, social support, short duration of living in a suburb, having no regret at leaving a suburb, sole parenthood, and mother’s country of birth and expectation of parenthood [19,23]. We have previously proposed that our findings are consistent with group-level socioeconomic deprivation, neighbourhood environment, social capital and ethnicity diversity causing postnatal depression and other adverse perinatal outcomes [19].

**Figure 1:**
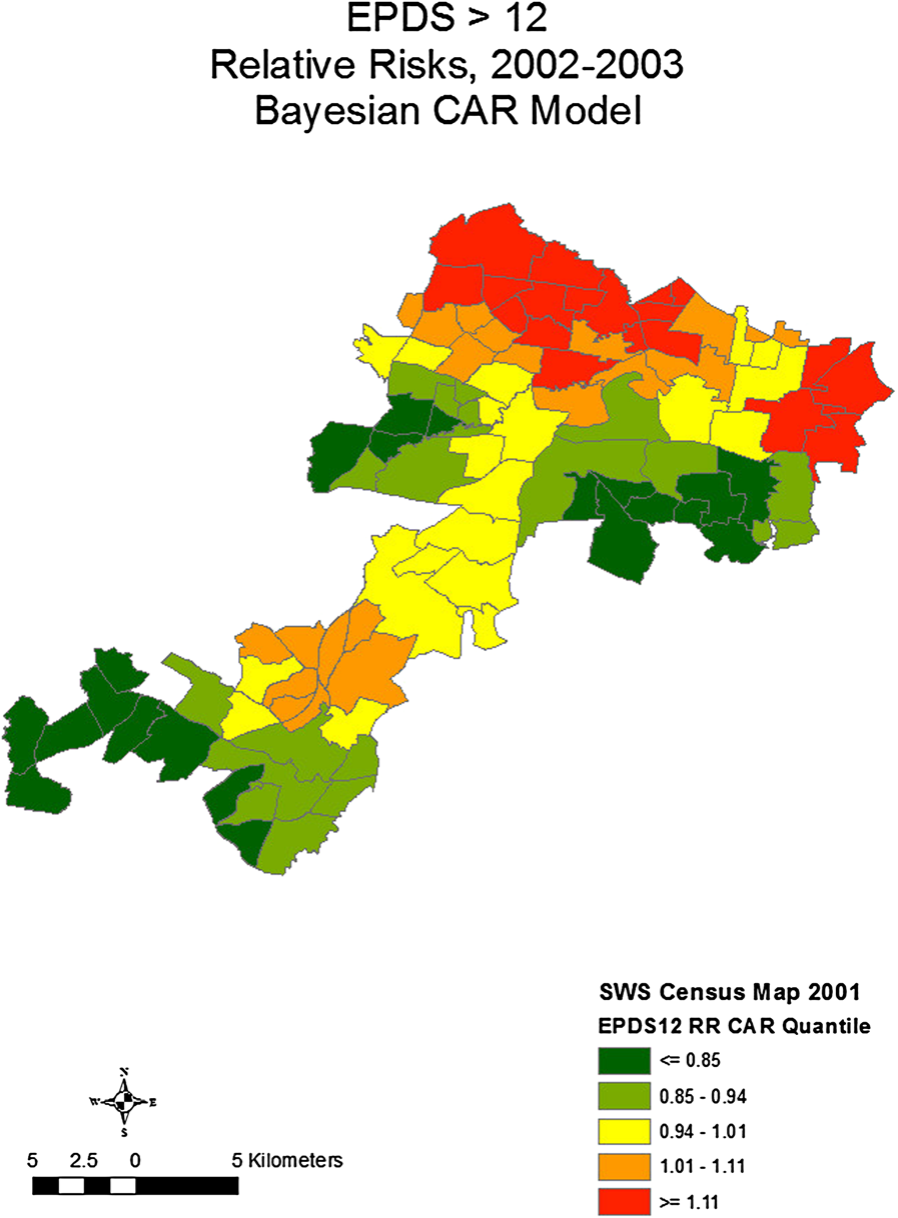
Bayesian CAR relative risks of EPDS > 12.

We have undertaken a multi-level mixed method research programme to inform the building of a theory of neighbourhood context, depression and the developmental origins of health and disease [24]. A non-linear principal component and regression analysis identified that social exclusion, infant behaviour, migrant social isolation and maternal expectations independently predicted postnatal maternal depressive symptoms [25]. Our suburb-level spatial studies identified clustering of postnatal depression in regions known to be socially disadvantaged and with high rates of migrant mothers (Figure 1) [26]. Subsequent ecological spatial and linear multivariable regression studies found that measures of social disadvantage, ethnic diversity and weak social networks were consistently associated with higher rates of depressive symptoms (EPDS >9) [27].

Social and physical environmental adversity and measures of social capital have previously been found to be associated with maternal stress, and poor pregnancy outcomes, including: prematurity, low-birth weight and infant mortality [28,29]. There is also increasing evidence from multilevel regression studies for an association of area-level economic deprivation and individual level mental health outcomes [30-32]. The evidence for a link between social capital and mental health has been less convincing. A systematic review undertaken by De Silva and colleagues [33] concluded that current evidence was inadequate to inform the development of specific social capital interventions to combat mental illness. The authors also observed that "individual and ecological social capital may measure different aspects of the social environment" [33].

A recent criticism of social epidemiological studies, and multi-level studies in particular, has been a paucity of theory [34-39]. Kaplan stated that "perhaps nowhere is the need for social epidemiological theory more apparent than in the study of "place" effects on health [39]. One of the main problems in the study of neighbourhoods and health is the lack of development of theories about plausible social, psychological, and biological links between specific features of the neighbourhoods and specific health outcomes [40]. Shankardass and Dunn [41] have recently argued that social epidemiology has been proficient in describing patterns in neighbourhood inequalities but has been less capable at fostering an understanding of how these effects relate to the social mechanisms of causation. They suggest using a critical realist approach to identify "explanatory" mechanisms.

The aim of the qualitative study reported here was to: 1) explain the observed clustering of postnatal depressive symptoms in South Western Sydney; and 2) identify group-level mechanisms that would add to our understanding of the social determinants of maternal depression. The paper aims to move beyond our previous description of individual-level predictors of depressive symptoms to explain how neighbourhood context influences maternal depression.

## Methods

### Methodology

Critical realism provided the methodological underpinning for the study. For critical realists, causation is not solely based on observed regularities in data (i.e. correlations or regression studies) but also on identifying the underlying causal mechanisms and how they work. Critical realist methodology seeks to identify the causal mechanisms that produce the observed or experienced phenomenon measured by empirical research studies. These causal mechanisms often cannot be directly, or empirically, observed and the task in explanatory research is to find the mechanisms that produce the actual phenomenon and to understand the interplay between them and what produces the outcomes or observed events.

The qualitative study reported here was part of the emergent phase of a critical realist Explanatory Theory Building Method consisting of three phases of research: 1) emergent phase; 2) construction phase; and 3) confirmation phase [24]. The emergent phase of theory building moves from the empirical observations and interpretations of the persons involved towards the development of theoretical concepts. The qualitative methodology chosen follows the theory building approaches described by Miles and Huberman [42] and Glaser and Strauss [43]. Miles and Huberman [42] describe an approach that starts with an organising framework for the codes derived from prior knowledge. In this emergent phase of study we predominantly followed the "grounded" approach first described by Glaser and Strauss [43] and used minimal "preset" codes. For the second stage of analysis, a conceptual mapping approach was used to develop causal networks as described by Miles and Huberman [42], pp 151-165.

### Setting

Australia has a stable liberal democratic system, is a highly developed country with the world’s 12^th^ largest economy and the fifth-highest per capita income. The Commonwealth of Australia has six States and two territories. Sydney is the capital city of the State of New South Wales (NSW) on the east coast. In 2010, the greater Sydney metropolitan area had a population of approximately 4.6 million people. The study area is four local government city councils in South Western Sydney. There is universal access to a taxation funded maternal and child health system which includes a universal postnatal home visiting service. Paid maternity leave was introduced in 2011.

The study area has a diverse multicultural population with 28.4% of the population born overseas compared with 17.8% for the rest of NSW. Twenty percent of infants are born to women from South East, North East or South Asia, and approximately one quarter of Sydney’s indigenous population live in South Western Sydney. South Western Sydney is an area of substantial social disadvantage, and has lower education attainment and lower income levels then other parts of NSW. As noted above we have previously reported on clusters of postnatal depression in this region and identified two statistically significant clusters of postnatal depression in northern communities within the study region.

### Sample selection

The focus groups were naturally occurring groups consisting of participants of existing mothers groups. The mothers groups were established by child and family nurses for mothers (and fathers) of newborn infants. Three mothers groups, with 3-7 members, were purposefully selected from: a) a community with medium to high density housing, low socio-economic circumstances and high numbers of overseas born mothers, b) a community with predominantly single dwellings, average socio-economic circumstances and average socio-economic circumstances, and c) a community with predominantly single dwellings, low socio-economic circumstances and high numbers of overseas born mothers. The three communities were selected to ensure that participants were from communities that had been identified as having both high and low rates of depressive symptoms. The participants were a mixture of: European, Greek, Middle Eastern and Chinese parents in their late 20s or early 30s. One participant was a father. All participants spoke English and interpreters were not required.

Selection of participants (n = 8) for practitioner interviews was also by purposeful sampling. Potential participants were identified based on: gender, local government area where they worked, experience with population subgroups, professional or industry background, and emerging concepts. Two participants were from non-government agencies with experience working with migrant mothers, five were child and family health field workers with experience working with mothers from a range of ethnic and social backgrounds and one participant was an experienced child public health practitioner. Interview guides for the initial interviews were as for the focus groups. The questions asked changed as analysis was undertaken and conceptual theory emerged.

### Interview process

Women in the selected mothers groups were invited to participate in the focus groups. Coercion was avoided by using Child and Family Nursing Services to invite the participants. Participation was voluntary, the purpose was made clear, questions were invited, benefits explained and signatures of both the researcher and participant obtained after there was agreement to participate. Focus group members knew each other and anecdotal feedback indicated that participants appreciated the opportunity to discuss postnatal depression. The participants were asked (and signed) to keep all details of the focus group participants and discussion confidential. To reduce the influence of investigator-participant interaction on the research process the focus groups were facilitated by a research assistant. The focus groups ran for approximately one hour. The discussion points included: what is depression and why do some women get it during pregnancy, why there might be more or less depression in some suburbs, what the characteristics of those suburbs might be, and whether there are factors at a city, state and national level that might increase or decrease a mother’s depression. Based on emerging themes from the first two focus groups, the third focus group was asked whether communities with high numbers of ethnic groups were likely to have higher rates of depression. A medical practitioner was available to discuss personal concerns and answer questions. Handouts on postnatal depression were given to participants at the end of focus group sessions.

The practitioner interviews were conducted by the first author. Coercion was avoided by managing the process through a "third party". The same open questions were used as for the focus groups. The focus group and practitioner discussions were all conducted in English, notes were taken, including general information on the setting and participants, and digitally recorded.

### Data analysis

Data analysis was conducted by the first author and reviewed by the second author. Open coding was the predominant approach taken to initial coding [44,45]. Interview transcripts were coded line-by-line and paragraph-by-paragraph. Each "incident" was coded into as many categories that it might fit during the early stages of concept and category generation to enable maximum emergence of patterns and relationships [46]. A second cycle of coding was undertaken where the most frequent or significant initial codes were used to develop categories that were more selective and conceptual [44], p 57. The coding process was considered finalised at the point when there was theoretical saturation. The data coding, memos and analysis was supported by use of Atlas ti 5.0 software.

A conceptual mapping approach was used to develop causal networks as described by Miles and Huberman [42], pp 151-165. Causal networks display the most important concepts and processes and the relationships among them. The identification of "important" emerging concepts for focused analysis was based on a) density of coding to that concept or closely related concepts, b) density of interrelationships and connections among codes, and occasionally c) cognitive reasoning. Decisions regarding the nature of the theoretical links among codes was based on a) information provided by key informants and parents, b) prior knowledge of empirical literature, c) supporting information arising from concurrent literature reviews and quantitative studies, and d) abstract reasoning. The conceptual and categorical coding of data required abstract reasoning and the constant comparative method described by Glaser and Straus [43].

### Ethical matters

The study obtained ethics approval from the University of New South Wales Human Research Ethics Committee. As discussed above, coercion and disempowerment of focus group participants was avoided by managing the process through the auspices of "third-party" mothers group organisers. Participation was voluntary, the purpose was made clear, questions were invited, benefits explained and signatures of both the researcher and participant obtained after there was agreement to participate. The "mothers group" participants were deliberately not drawn from currently stigmatised communities or neighbourhoods. The focus group members knew each other and signed a confidentiality form declaring that all details of the focus group participants and discussion would be kept confidential. The privacy and anonymity of the participants was maintained and the data will be destroyed after 7 years. The information regarding the study also included information that had previously been distributed as part of national and local perinatal depression initiatives.

## Results and discussion

The qualitative research results are reported thematically below. Confidentiality is preserved for comments and quotes by only identifying them as mothers group or practitioners. While practitioners talked freely about the importance of community or neighbourhood level factors the mothers were less certain. Mothers generally believed that individual level factors were more important because "each individual that is in that suburb is in different circumstances anyway".

We have identified seven theoretical concepts that might explain the observed spatial clustering of maternal depression. They were: Community-level social networks; Social capital and Social cohesion; "Depressed community"; Access to services at the group level; Ethnic segregation and diversity; Supportive social policy; and Big business. The concepts that emerged from the analysis were not distinct. There were codes that contributed to more than one concept. For example a code such as "maternal isolation" was taken from "if you don’t have a big network you might just feel isolated". "Maternal isolation" was a property of "depressed communities" but was protected against by "community-level social support networks", "social capital and cohesion", "access to services" and "supportive social policy". Its relationship with "ethnic segregation or diversity" and "big business" was more complex.

### Community-level social support networks

Support was seen by mothers and practitioners as an important protection against maternal depression. Support had a number of sub-categories including partner support, family support, emotional support, and access to services. Social support networks included family members and friends and were thought to operate mainly at the level of the individual mother. Some felt, however, that an element of social support might operate at the group level and be stronger in some communities than others. One mothers group, for example, felt that wealthy suburbs would have more support and that was because they have less to worry about. Others felt that the support was related to access to mothers groups, services and other resources in the community.

All three focus groups, and some practitioners, identified community safety as impacting on support networks and maternal isolation. One group spoke of the isolation that resulted from the fear of going to the park or places where they might meet others. This link between the categories of "support", "social network" and "isolation" was explained by one mother as

"Sometimes may be there is lack of support, if you don’t have a big network, you just might feel isolated".

There also seemed to be physical aspects of communities that might contribute to social support networks. One mothers group talked about the importance of parks and playgrounds where they would come across other parents. The importance of town centres was raised by both practitioners and parents as places where mothers can meet each other. Access to the community shopping centres was seen by one practitioner as being important for social networks and "to feel linked". There was also comment that the development of large shopping malls had drawn people away from local community centres and had had a negative impact on local social networks. The lack of public transport from suburbs to the large community centres contributed to isolation and the lack of social networks.

### Social capital and cohesion

**Figure 2:**
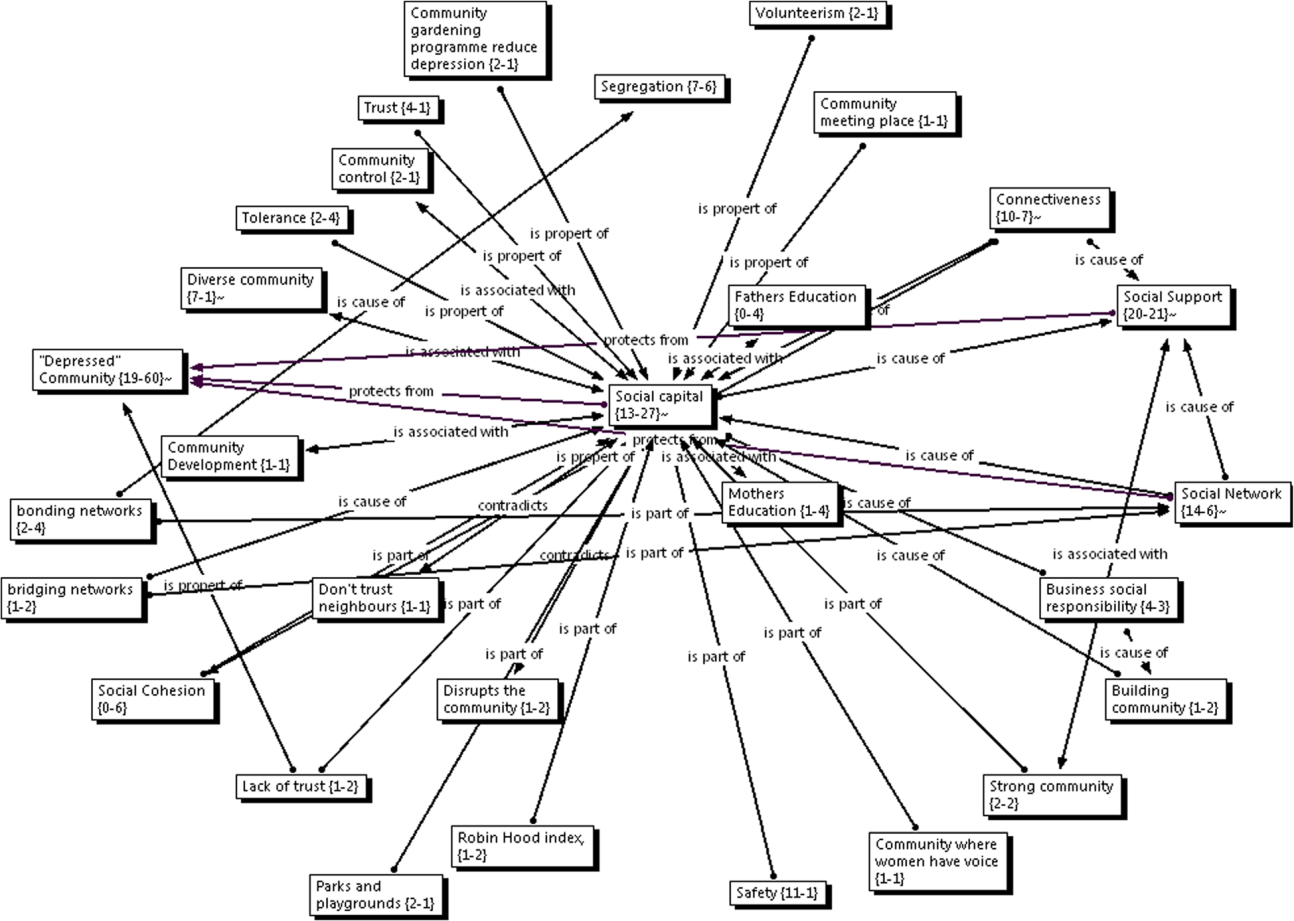
Focused Network: Social Capital.

The category "social capital" (Figure 2) emerged from the practitioner interviews who spoke of it principally in relation to the related categories of "social networks", "social support" and "connectedness". Social capital was also discussed by those practitioners in relation to negative terms such as "depressed community", "segregation" and "marginalisation". Data from mothers groups did not identify this term as a category. One practitioner noted that:

"there are higher rates of child abuse amongst poor populations that also have low social capital".

A link was drawn to maternal perinatal emotional health and associated physical and emotional abuse. It was thus speculated that where there was no social capital there would be marginalisation and no social support networks. The same key informant drew links between high social capital and "inclusiveness as a culture" as being not that much different to a homogenous population where marginalisation is not tolerated or encouraged. Thus a community with strong social capital would have social support networks and no marginalisation.

Social capital was further described as being affected by "levels of the size of the gap between rich and poor" with research showing a "consistent linear relationship of bigger the gap between rich and poor [the] lower [the] levels of social capital". The implication of this idea is that there would be low social capital where there is inequality. Other practitioners, however, talked of low social capital existing in disadvantaged "depressed communities", and in communities where there was segregation or marginalisation of people along ethnic and religious lines.

Several practitioners spoke of "building community" and the importance of "investing in the people and the community to make the community theirs". They spoke of the important role that "big business" and community leaders had in investing in the community. Physical attributes described as contributing to social capital included parks, shopping centres, play grounds, community meeting places, and community gardens. One practitioner spoke of the importance of local football teams. The practitioner noted that

"we had a wonderful system of football teams in Sydney and it is completely disrupted .. it got nationalised and that regional cohesion got lost … The football teams should be investing in the community".

A related category was connectedness. Several practitioners spoke of the positive role that community connectedness might play and one spoke of "belonging to community" which we have interpreted as being a part of connectedness. Connectedness was seen as a protective or resilience factor that might enable a "mother to ride out depression better than someone in another neighbourhood who didn’t have access to that good connection to community".

As observed by Kawachi and colleagues [47] there has been a lack of consensus concerning the definition of social capital with at least two different schools of "social capital". The authors observe that there is currently contention as to whether social capital should be conceptualised as social cohesion or as resources imbedded in social networks. This confusion was evident in the study reported here with practitioners discussing both concepts interchangeably. By contrast, mothers spoke mainly of the importance of social networks.

The debate is well articulated by Carpiano [48,49] who compared two theories of social capital as advanced by Putman [50-52] and Bourdieu [53,54]. Carpiano [49] argues for the separation of what he calls the: 1) structural antecedent factors (i.e. socio-economic conditions, residential stability, income inequality), 2) social cohesion (connectedness and values), 3) social capital (social support, social leverage, information social control, neighbourhood organisation participation), and 4) social capital outcomes.

Such an approach might be consistent with the emergent findings reported here. We propose, therefore, that we adopt the theoretical concepts advanced by Carpiano. The theoretical concept "community-level social networks" will be called "community-level social capital" and the second theoretical concept be called "social cohesion" with the attributes of connectedness and values.

### "Depressed" community

**Figure 3:**
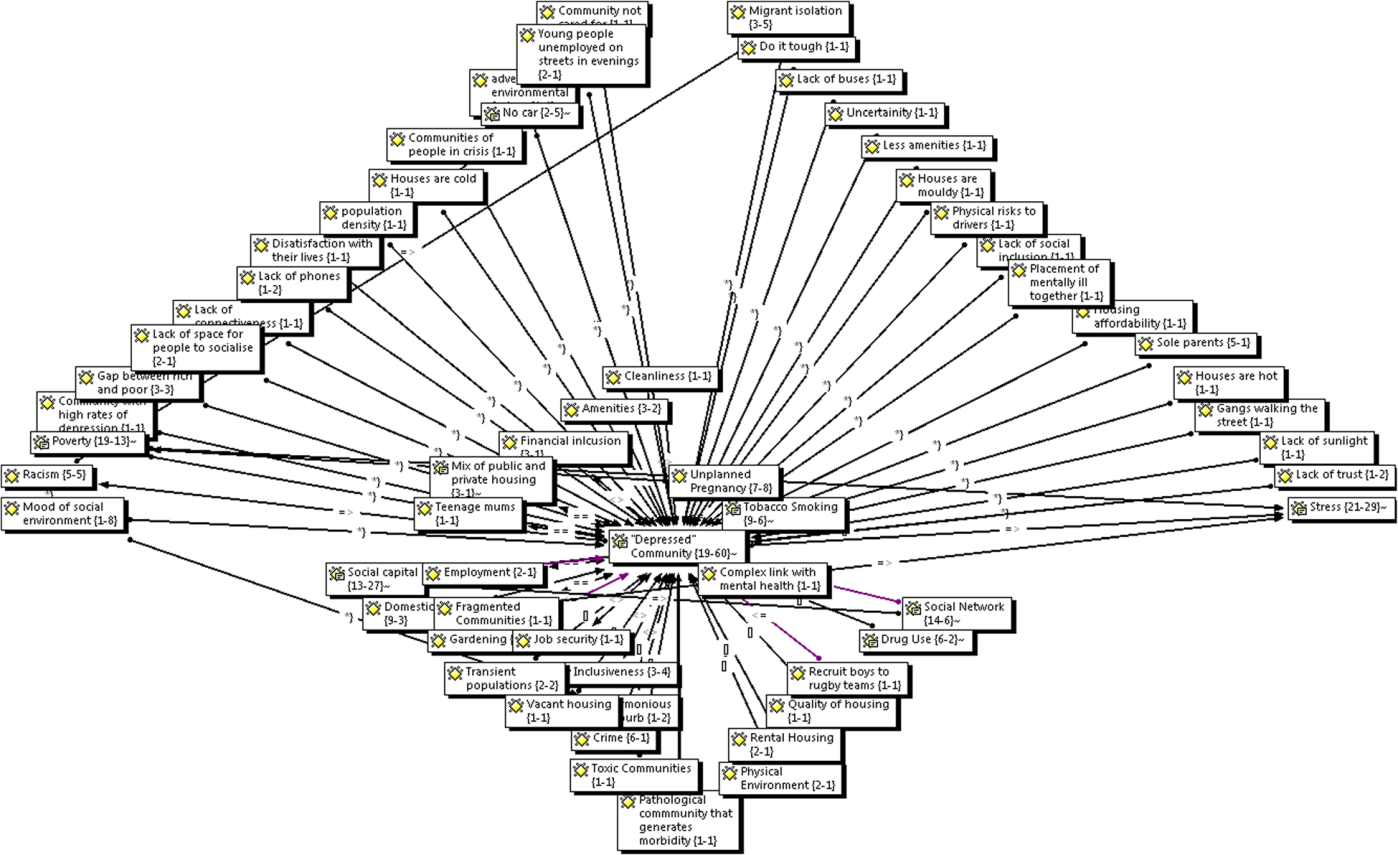
Focused Network: Depressed community.

A strong theme emerging was the image of a "depressed" community (Figure 3) where there might be elements of both physical and social environmental deprivation. The category "depressed" community had a large number of sub-categories that together give a fuller picture of a depressed community. They include: low family income, high rates of tobacco smoking, high rates of unplanned pregnancy, high rates of sole parenthood, community not looking cared for, high rates of crime, domestic violence, gangs walking the street, lack of buses, lack of space for people to socialise in, fewer amenities, houses that are either too cold or too hot, lack of telephones, rental and public housing, poor quality housing, lack of trust, racism, migrant isolation, transient populations, vacant housing, young unemployed people on the streets in the evenings, high numbers of mentally unwell residents, high population density, lack of connectedness, lack of social inclusion, and a depressed mood in the community.

One practitioner gave a colourful description of disadvantaged communities that they had worked in –

"streets are barren, there aren’t people walking in the streets … youth walk around in gangs, so mums won’t let [children] walk, … houses are poorly kept … gardens overgrown … lounges on the front veranda".

When mothers were asked to describe a community with high rates of depression they described such a community as being "grubby", "having transport problems", "not being looked after", "people doing it tough", "being in the middle of nowhere and nowhere to go to", "isolation", "new immigrants to the country … don’t have established social networks", "environments that is not probably not healthy for your child", "there is violence", "where you can’t get to, say, a mothers’ group, don’t have family close by", "you can’t get out and about to shops", "stuck in the middle of nowhere … being cooped up in a house with a kid 24/7", "violent people then you choose not to go the park … you don’t want to especially now you have a baby, you want to keep him safe … then you are isolated".

The picture given by these mothers is of a "grubby" poorly cared for community with violence, poor transport and resulting isolation. The emphasis was on isolation -

"if you are in the middle of nowhere and nowhere to go to".

Both mothers and practitioners spoke of the importance of transport. Communities were described as having an irregular bus service because there was an expectation that people would have a car but

"there is only one car in the family, the partner in the house has the car. The woman is at home. It is an effort to pack babies and kids up and take them out and do shopping and do all those things".

The lack of transport is related to poor access to services and amenities. We have identified access to services as a major emerging theme that is discussed further below.

### Access to services at the group-level

Access also emerged as a major concept at the group-level with related sub-concepts that include: access to medical care, access to shopping centres, access to baby checks, access to child care, access to services, location of services, access to books, information, transport, coordination of services, services not talking to each other and courage to make a call for help.

Mothers did not use the phrase "access" which was generally used by practitioners. Mothers did, however, talk of transport problems often in relation to comments on isolation –

"I think transport problem is significant, it is hard to get out and about, I suppose there is isolation".

When talking about communities with low rates of depression mothers linked this to having access to information –

"knowing where the community nurse is and that sort of thing"

"I guess it is more to do with plenty of supply of resources, if you need them, just having in your mind that it is there".

Mothers spoke of the importance of the services provided by midwives including services for postnatal depression. Having, for example, someone to -

"look over a mother .. especially if there is not a lot of support from the partner at least there is someone there to identify that they might have postnatal depression".

The home visiting nurse services and mothers groups were considered to be important services to access. Home visiting was clearly a way by which mothers receive support from health services. Several mothers spoke of the help and support given by the community child health nurses. Mothers also spoke of the importance of antenatal classes and the nurse home visits to their involvement with mothers groups which provided

"those connections from the beginning to continue all the way through".

The accessing of services was seen by both mothers and practitioners as being particularly difficult for new immigrants who did not have established networks, and had difficulty communicating their needs and accessing interpreter services.

Both mothers and practitioners described the buffering role that social networks, connectedness and access to services would have on mothers isolated in those communities. Access to community facilities and services was identified by mothers as protecting mothers from postnatal depression. Hutchinson and colleagues [55] found a buffering impact of social services on maternal (and infant) psycho-social and psychological processes. Recent studies have demonstrated the effectiveness of support-focused interventions by social services. The efficacy of home visiting and telephone based support for postnatal depression has recently been confirmed [56-58]. Broader social service intervention that are often described as "joined-up Government" may also have benefits [59].

### Ethnic segregation or diversity

**Figure 4:**
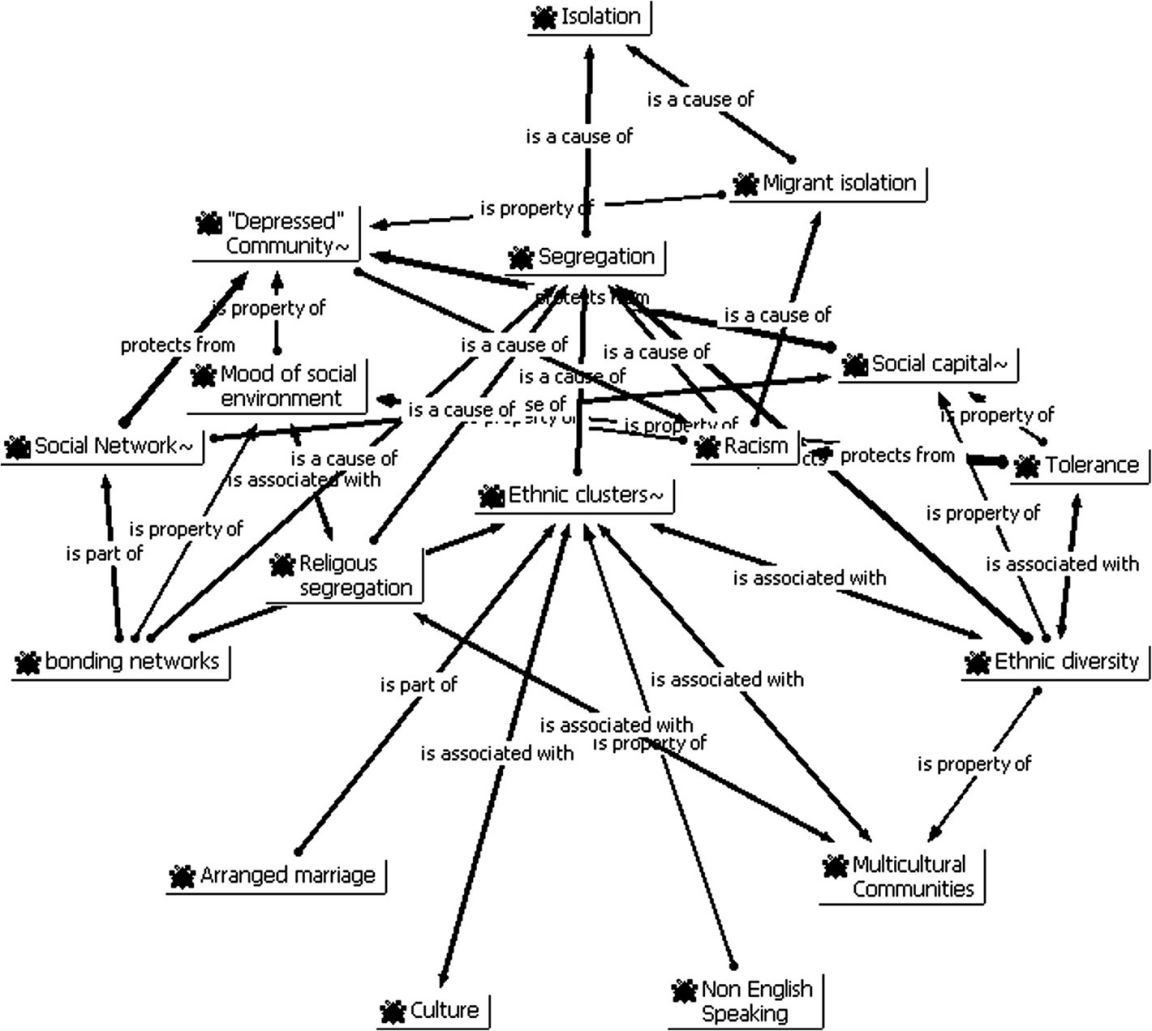
Focused Network: Ethnic Segregation.

Practitioners identified a link between social cohesion and ethnic segregation (Figure 4). One practitioner when speaking of social cohesion spoke of the divide ..... between Arabic and Vietnamese groups. They spoke of further divides within the Arabic speaking community with Arabic speaking families coming from 22 nations. For them, social cohesion was around

"looking at where are the glue points where we can glue people together to form the fabric of the community".

In contrast to the ethnic segregation that they had experienced in one suburb, the same practitioner spoke of integration in another suburb where Muslim women were participating in a multicultural group rather than their Muslim women’s group. She quoted the mothers as saying

"but we want to be part of our community. If we go there we are not part of the community, we are just other Muslim women. By coming here we are part of the community. We want to know other people".

By contrast in another area, some ethnic groups did not want to meet with others because they didn’t like the way women from other cultures parented.

"One of the key issues, why they were getting together, was around parenting, and in a sense in a negative way it was this strong sense of racial arrogance that was coming out in these young women. I was a bit blown away from having this experience with these other women to having this experience with these women who did not emphatically want to relate to other women of another race".

With that different experience among the culturally diverse communities, the practitioner wondered whether it was related to the size of the various ethnic populations.

"What I found startling was, what we find in [community] is there is a huge Vietnamese community and a huge Arabic community. Is it because they are so large that they end up becoming quite separate? Whereas in the other two, [communities], does the salt and peppering of the multinational nature of it mean that there is a greater degree of integration?"

Mothers in the focus groups also highlighted the possible role of ethnic clusters. One mother when asked why some women might get more depressed than others wondered if it may be because there are clusters of people from different cultures living in those communities.

One mothers group drew attention to the role of language and culture noting that:

"may be some people don’t speak English, we have a special language group… if they don’t know they just isolate… may be some culture they don’t know how to have friends … sometimes we can’t mix with other people, you have to move with the same religion [or] culture."

Another mothers group felt that some communities might have more maternal depression because there were newer immigrants who might not have "family or support" or "an established social network".

As noted above, practitioners identified a link between social cohesion and ethnic segregation. Divisions between Arabic and Vietnamese groups were noted with a tendency for larger ethnic groups to operate within their ethic group. By contrast in more diverse communities migrant women participated in multicultural groups because "we want to be part of our community". It was also observed that with large homogenous ethnic groups, mothers did not want to meet with mothers outside their ethnic group because they didn’t like the way women from other cultures parented.

There is an extensive literature concerning ethnic segregation. Kramer and Hogue [60] identified 39 studies that tested the association between segregation and health outcomes. Segregation was associated with poor pregnancy outcomes and increased mortality. Several studies, however, reported health-protective effects of living in clustered black neighbourhood after controlling for social and economic isolation. In their review of 15 such studies, Acevedo-Garcia and Lochner [61] found that none of the studies explored why and how residential segregation influences health, tested specific pathways or developed multilevel models. They hypothesized that segregation had an indirect effect on health outcomes operating through multiple mediators [61].

There have been significant waves of recent migration to South Western Sydney and explanations for the clusters of maternal depression may lie in an eco-cultural framework described by Berry and colleagues [62]. The authors described antecedent mechanisms related to population-level characteristics such as the ecological and socio-political context. They viewed the intervening processes as genetic transmission, cultural transmission, ecological influences and acculturation [63].

### Supportive social policy

A theme emerged in relation to what we have called here supportive social policy. Practitioners and mothers both spoke of the importance of social policy matters such as financial support for families, maternity leave, and free child care. One practitioner spoke often of comparisons with the Scandinavian countries while others talked of matters related to local council social planning.

Mothers spoke of the importance of financial help as a way of decreasing postnatal depression. This was seen as the national Government’s responsibility. A related question was whether Medicare (universal health insurance system) was available to support mothers with postnatal depression. There was concern expressed regarding the means testing. Lack of government financial support was linked by one mother to stress. She stated that

"usually by the time you have a child you are living beyond your means anyway".

Practitioners supported the view that financial support was important. One practitioner linked financial inclusion and food security with the

"ability to be in control of your life".

Another practitioner felt that there needed to be a shift in employment patterns so that people did not have to travel so far to get work. They suggested there be more small business parks so that people did not need to travel to the city for work. Another practitioner spoke of the active work of local government councils … with active investment in community development initiatives such as playgroups, community parks and transition to school initiatives. This practitioner felt that there was a big difference between the activities undertaken by the [one] local council and others in the region. One practitioner felt that some local councils

"become so immersed in the politics and bureaucracy [that] their ability to partner purposefully and openly is limited by that political nature of the beast".

They drew a link with lack of social cohesion and noted that some local government councils where investing in community work while others were not.

The mothers were clear that income support, nurse phone calls, and mothers groups all contributed to supporting them when they felt "out of control" or depressed. An unexpected finding during the research was the limited number of mothers groups available. This was surprising considering the potential that they have as both being protective and supporting mothers with depression and other difficulties. The answer may lie in the nature of previous NSW government policy which had focused on the delivery of a single universal home visit by child and family health nurses. This highlights the important role that social policy plays in influencing the situation for mothers vulnerable to depression.

### Big business

**Figure 5:**
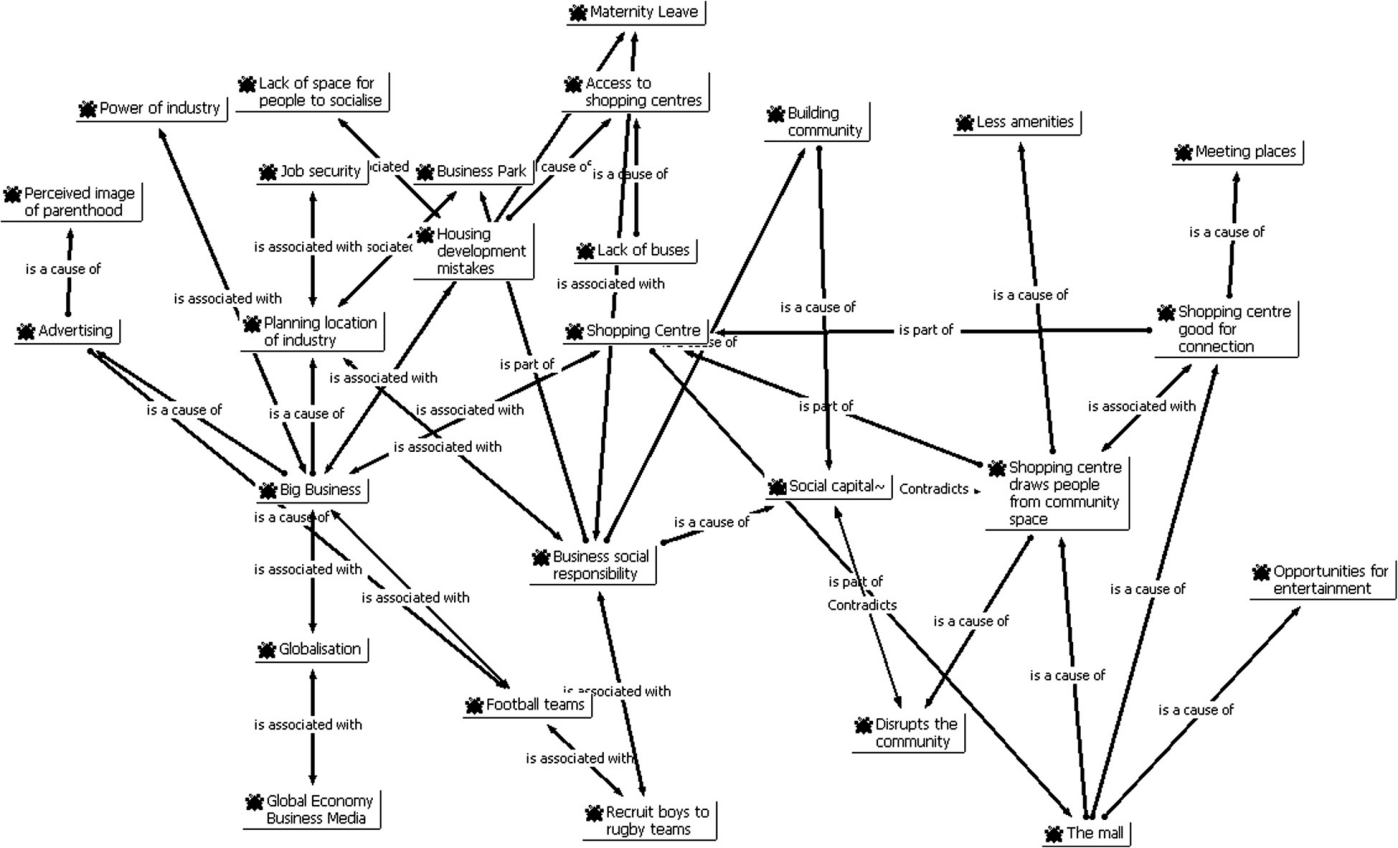
Focused Network: Big Business.

The concept of "Big Business" (Figure 5) emerged from the data as a concept related to the national, regional and local activities of corporate business. Big businesses were seen as important players in the provision of support to communities and mothers. Big business and media were variably described as having both positive and negative influences. The impact of sports franchises on local communities was discussed by one practitioner who felt that an important contribution to community had been removed when sports codes had been "nationalised". Another practitioner spoke of the impact that shopping malls were having on small community centres where both people and services were attracted away from small communities to the larger town centres.

"we are drawing people out of our public spaces which in fact creates a sense of safety and community to a closed environment which means you are only seeing people when you shop, you are only feeling safe in those shopping centres .. They are not going to a local park because the Council has locked it up."

While this was seen by some as having a negative impact, others felt that the new shopping malls provided opportunities for mothers to network and to access services.

"The owners of large shopping malls have the ability to make both positive and negative impact."

One practitioner was clearly upset at the limited involvement that local shopping centre owners where having in their community and spoke of the impact on children’s play space

"we should actually be building the shopping centres around the playground making the playground in the shopping centre. … Then what are we doing, we are actually taking away the ground space from the children and saying the place where you play is the shopping centre".

The large shopping centres were also seen as placing financial pressure on families

"those big shopping centres are the place to meet people. The focus of the eye is drawn a bit like the dot on the blank wall, the eye is drawn to the spending of money as the way to happiness and connecting to people".

Local council community activities were also impacted by big business. An example was given by one practitioner of the failure of local government to support the development of a community gardening project, electing instead to lease the land to a large fast food franchise.

The impact of the large shopping malls on local communities was seen as both having positive and negative impacts on mothers as it provided meeting places for mothers but also "depopulated" local shopping areas and parks. It was clear from this study that practitioners felt that Big Business had the "power" to influence local politicians. One example given, related to the building of "fast food" outlet on land that community members wished to use as a community garden.

Our findings are consistent with the "McDonaldization" thesis advanced by Ritzer [64], p 457. Ritzer describes this as "the process by which the principles of the fast-food restaurant are coming to dominate more and more sectors of society". Ritzer describes the process as being delineated by efficiency, calculability, predictability, control through technology and "irrationality of rationality". The later process inevitably leads to dehumanisation of jobs, settings and circumstances [64], p 459 with impacts on local neighbourhoods and communities.

During the course of this study, South West Sydney experienced the impact of global economic mechanisms with closure of large businesses and loss of employment. Big Business elected to move to other countries and jurisdictions. As a result the NSW Government budgets were affected leading to impacts on urban development, social services and maintenance of essential infrastructure. Strong global economic forces are also responsible for movement of migrants and refugees to South Western Sydney. Some are economic migrants while others are from past and present conflicts. The region is vulnerable to these global economic fortunes and the landscape of local communities are influenced as much by the planning decisions of Big Business as they are by local planners and policy makers.

### Limitations and strengths

Mothers were interviewed at a mothers group. Consequently those mothers had experienced support from a mothers group. They placed great significance on the importance of the mothers groups for establishing and maintaining support networks. One mother noted that support networks may include family, friends and neighbours. The mothers who attended the groups were not themselves from socially disadvantaged backgrounds and few mothers were from non-English speaking backgrounds. The number of mothers groups interviewed was limited in this study to three which may limit generalizability. Despite these limitations the qualitative data was rich and contributed significantly to both theory generation and later theory construction. It was notable that the findings from this qualitative study are similar to those found by Beck in both her original phenomenological [65] and grounded theory [66] studies and in her later meta-synthesis [67].

The qualitative (intensive) methods used in this study included interviews and focus groups with qualitative analysis. The strength of these approaches is their ability to provide explanatory power to the analysis [68] and thus the identification of possible causal mechanisms. The quality of such intensive or qualitative studies is assessed using criteria that differ from those used for extensive quantitative studies [69-72]. The criteria proposed by Kitto and colleagues [69] were used to assess the quality of the current study. The procedural rigour was made explicit through clear articulation of the ontological and epistemology position informing the study which then informed the emergent and explanatory study design, methods of data collection and analysis. The sampling techniques used were purposeful and sought to include subjects from different communities, and ethnic backgrounds.

Interpretive rigor was enhanced by integrative and synergistic use of both quantitative and qualitative data. The theoretical literature was reviewed following the emergence of conceptual model to enhance theoretical sensitivity and the presentation of the results included extensive use of participants’ quotes to support interpretation. Reflectivity was maintained by acknowledging our theoretical perspective and prior knowledge. Distance from the subjects was achieved by intentionally engaging a research assistant to conduct the focus groups. The ethical and local political aspects of the research were described including the ethics approval. It is uncertain to what extent the findings of the intensive study will be transferrable. The intention of this aspect is not generalisability but rather explanation. The explanations provided were consistent with those found in other studies suggesting that some transferability might be possible.

We have used here the meta-theory of critical realism for the generation of causal explanations in social epidemiology as a response to the criticisms put forward by Muntaner (1999), O’Campo [36] and Raphael [38]. The development of realist methodologies in epidemiology and population health is relatively new although advances have been made in policy and program evaluation [73] and evidence based reviews [74]. As a meta-theory, critical realism seems to be ideally suited for social epidemiology theory building and testing. Qualitative methods for confirmatory studies are well supported by critical realism [68,75] and realist approaches are gaining credibility in relation to evidence-based policy and programme evaluation [73,74,76]. From a critical realist perspective, quantitative modelling is also seen as useful to test proposed explanations [77].

### Implications for further research

#### Factor analysis

The codes and concepts emerging from the qualitative analysis were not distinct with significant overlap between social capital, social cohesion and ethnic segregation. Some degree of overlap with these theoretical constructs is to be expected especially given the current theoretical debates discussed earlier. In critical realist terms, factor analysis may assist in further defining measurable empirical indicators and their associated latent variables [78].

#### Case studies

Missing from this study has been an intensive study of the experiences of depressed mothers. The qualitative findings are consistent with previous qualitative studies but further confirmation and understanding of the phenomenon can be gained from conducting case studies with mothers who have, or are experiencing postnatal depression. Case studies are consistent with critical realist evaluation and confirmation approaches [79]. Such case studies could be used to examine the propositions arising from this study. In particular, case studies should include migrant women, young unsupported mothers, mothers in depressed neighbourhoods and those living in suburbs with strong aggregated social networks.

#### Social network studies

Social networks were identified in this study as playing an important buffering role against stress and depression. Questions arose in the study regarding the role of bridging, bonding and linking networks particularly among migrant mothers. Social network analysis is one research method that can assist with understanding these networks. Typically, social network analysis relies on questionnaires and interviews to gather information about the relationships within a defined group. The purpose of a social network analysis would be to explore in an intensive manner the nature of the social ties among depressed mothers with a focus on differences across ethnic and migrant groups.

#### Concept mapping

O’Campo and colleagues [80] recently described a qualitative study that identified pathways by which neighbourhoods affect mental well-being. That study design is well suited for confirmatory and follow-up studies. For that study the authors used concept mapping [81]. Concept mapping is a "structured process, focused on a topic or construct of interest, entailing input from one or more participants that produces an interpretable pictorial view (concept map) of their ideas and how these are interrelated". Both qualitative and quantitative methods are used to create a visual display of how the participants and the group conceptualise a given topic [80].

## Conclusion

For this study we took as our starting the point the proposition that maternal stress and depression adversely impact on the developmental origins of health and disease and that the tendency of stress to cause depression is almost certainly conditional on personal characteristics and social and cultural context. We had previously proposed that the findings of our individual level studies of maternal postnatal depressive symptoms were consistent with group-level socioeconomic deprivation, neighbourhood environment, social capital and ethnic diversity having causal effects. Our suburb-level spatial studies had identified clustering of postnatal depression in regions know to be socially disadvantaged and with high rates of migrant mothers.

The aim of the qualitative study reported here was to: 1) explain the observed clustering of postnatal depressive symptoms in South Western Sydney; and 2) identify group-level mechanisms that would add to our understanding of the social determinants of maternal depression.

Using an open coding approach to maximise emergence of patterns and relationships, we have identified seven theoretical concepts that might explain the observed spatial clustering of maternal depression. The theoretical concepts identified were: Community-level social networks; Social Capital and Social Cohesion; "Depressed community"; Access to services at the group level; Ethnic segregation and diversity; Supportive social policy; and Big business. The theoretical concepts identified are tentative in keeping with our critical realist epistemology. It remains probable that community-level social networks, social capital, social cohesion and ethnic segregation are overlapping concepts representing closely related social mechanisms. As noted above, there is a lack of consensus regarding these concepts [47]. For quantitative operationalization, we used empirical indicators chosen to represent the identified qualitative codes [82]. These were analysed using exploratory factor analysis, ecological, spatial and multilevel regression [27,83]. The factor analysis latent variables were similar to the theoretical concepts emerging from the study reported here [27,82].

We postulate that these regional structural, economic, social and cultural mechanisms partially explain the pattern of maternal depression observed in families and communities within South Western Sydney. We further observe that powerful global economic and political forces are having an impact on the local situation. The challenge for policy and practice is to support mothers and their families within this adverse regional and global-economic context.

## Abbreviations

EPDS: Edinburgh postnatal depression; NSW: New South Wales.

## Competing interests

The authors declare that they have no competing interests.

## Authors’ contributions

JGE made the study designs, conceptualised the report, did the data analysis, data interpretation, and wrote the report. LAK and BBJ made critical and technical contribution to the study design, analysis and report writing. All authors read and approved the manuscript.

## Pre-publication history

The pre-publication history for this paper can be accessed here:

http://www.biomedcentral.com/1471-2393/14/47/prepub

## References

[B1] Gluckman P Hanson M The Developmental Origins of Health and Disease 2006 Cambridge: Cambridge University Press

[B2] Matthews S Meaney M Riecher-Rossler A, Steiner M Maternal Adversity, Vulnerability and Disease Perinatal Stress, Mood and Anxiety Disorders From Bench to Bedside 2005 Basel: Karger

[B3] Meaney M Epigenetics and the biological definition of gene x environment interactions Child Dev 2010 81 1 41 79 10.1111/j.1467-8624.2009.01381.x 20331654

[B4] Beck CT The effects of Postpartum Depression on Maternal-Infant Interaction: a meta-analysis Nurs Res 1995 44 5 298 304 7567486

[B5] Murray L Stanley C Hooper R King F Fiori-Cowley A The role of infant factors in posnatal depression and mother-infant interactions Dev Med Child Neurol 1996 38 2 109 119 8603778 10.1111/j.1469-8749.1996.tb12082.x

[B6] Martins C Gaffan E Effects of early maternal depression on patterns of infant-mother attachment: a meta-analytic investigation J Child Psychol Psychiatry 2000 41 6 737 746 10.1111/1469-7610.00661 11039686

[B7] Cogill S Caplan H Alexandra H Robson K Kumar R Impact of maternal post-natal depression on cognitive development of young children Br Med J 1986 292 1165 1167 10.1136/bmj.292.6529.1165 3085767 PMC1340177

[B8] Downey G Coyne J Children of depressed parents: An integrative review Psychol Bull 1990 108 50 76 2200073 10.1037/0033-2909.108.1.50

[B9] Gelford D Teti DM The effects of maternal depression on children Clin Psychol Rev 1990 10 329 353 10.1016/0272-7358(90)90065-I

[B10] Murray L Hipwell A Hooper R Stein A Cooper P The cognitive development of 5-year-old children of postnatally depressed mothers J Child Psychol Psychiatry 1996 37 8 927 935 10.1111/j.1469-7610.1996.tb01490.x 9119940

[B11] Cummings E Davies P Maternal depression and child development J Child Psychol Psychiatry 1994 35 73 112 10.1111/j.1469-7610.1994.tb01133.x 8163630

[B12] Sohr-Preston S Scaramella L Implications of timing of maternal depressive symptons for early cognitive and language development Clin Child Fam Psychol Rev 2006 9 1 65 83 10.1007/s10567-006-0004-2 16817009

[B13] Engle P Maternal mental health: program and policy implications Am J Clin Nutr 2009 89 3 963S 966S 10.3945/ajcn.2008.26692G 19176734

[B14] Wachs T Black M Engle P Maternal depression: a global threat to children's health, development, and behavior to human rights Child Dev Perspect 2009 3 51 59 10.1111/j.1750-8606.2008.00077.x

[B15] Wan M Sharp D Howard L Abel K Attitudes and adjustment to the parental role in mothers following treatment for postnatal depression J Affect Disord 2011 131 1–3 284 292 21349585 10.1016/j.jad.2011.01.009

[B16] Gavin N Gaynes B Lohr K Meltzer-Brody S Gartlehner G Swinson T Perinatal depression. a systematic review of prevalence and incidence Obstet Gynecol 2005 106 1071 1083 10.1097/01.AOG.0000183597.31630.db 16260528

[B17] Milgrom J Mendelsohn J Gemmill A Does postnatal depression screening work? Throwing out the bathwater, keeping the baby J Affect Disord 2011 132 3 301 310 10.1016/j.jad.2010.09.031 20952072

[B18] Buist A Austin M Hayes BA Speelman C Bilszta J Gemmill A Brooks J Ellwood DA Milgrom J Postnatal mental health of women giving birth in Australia 2002–2004: findings from the beyondblue National Postnatal Depression Program Aust N Z J Psychiatry 2008 42 66 73 10.1080/00048670701732749 18058446

[B19] Eastwood J Phung H Barnett B Postnatal depression and socio-demographic risk: factors associated with Edinburgh depression scale scores in a metropolitan area of New South Wales, Australia Aust N Z J Psychiatry 2011 45 12 1040 1046 10.3109/00048674.2011.619160 22017687

[B20] Kinderman P A psychological model of mental disorder Harv Rev Psychiatry 2005 13 4 206 217 10.1080/10673220500243349 16126607

[B21] Stone E Lin Y Quartermain D A final common pathway for depression? Progress toward a general conceptual framework Neurosci Biobehav Rev 2008 32 3 508 524 10.1016/j.neubiorev.2007.08.007 18023876 PMC2265074

[B22] Beck CT Predictors of postpartum depression: an update Nurs Res 2001 50 5 275 285 10.1097/00006199-200109000-00004 11570712

[B23] Eastwood J Jalaludin B Kemp L Phung H Barnett B Relationship of postnatal depressive symptoms to infant temperament, maternal exepectations, social support and other potential risk factors: findings from a large Australian cross-sectional study BMC Pregnancy Childbirth 2012 12 148 10.1186/1471-2393-12-148 23234239 PMC3556157

[B24] Eastwood J Jalaludin B Kemp L Realist explanatory theory building method for social epidemiology: a protocol for a mixed method multilevel study of neighbourhood context and postnatal depression SpringerPlus 2014 3 12 10.1186/2193-1801-3-12 24422187 PMC3888492

[B25] Eastwood J Jalaludin B Kemp L Phung H Barnett B Tobin J Social exclusion, infant behaviour, social isolation and maternal expectations independently predict maternal depressive symptoms Brain and Behavior 2012 3 14 23 23408743 10.1002/brb3.107 PMC3568785

[B26] Eastwood J Jalaludin B Kemp L Phung H Adusumilli S Clusters of maternal depressive symptoms in South Western Sydney, Australia Spat Spatiotemporal Epidemiol 2013 4 25 31 23481251 10.1016/j.sste.2012.11.001

[B27] Eastwood J Jalaludin BB Kemp L Phung H Neighbourhood adversity, ethnic diversity, and weak social cohesion and social networks predict high rates of maternal depressive symptoms: A critical realist ecological study in South Western Sydney Australia. Int J Health Serv 2013 43 2 241 266 10.2190/HS.43.2.d 23821904

[B28] Buka S Brennan R Rich-Edwards JW Raudenbush S Earls F Neighbourhood support and the birth weight of urban infants Am J Epidemiol 2003 157 1 1 8 10.1093/aje/kwf170 12505884

[B29] Morenoff J Neighbourhood mechanisms and the spatial dynamics of birth weight Am J Sociol 2003 108 976 1017 10.1086/374405 14560732

[B30] Fone D Lloyd K Dunstan F Measuring the neighbourhood using UK benefits data: a multilevel analysis of mental health status BMC Public Health 2007 7 69 10.1186/1471-2458-7-69 17477868 PMC1878475

[B31] Weich S Twigg L Holt G Lewis G Jones K Contextual risk factors for the common mental disorders in Britain: a multilevel investigation of the effects of place J Epidemiol Community Health 2003 57 616 621 10.1136/jech.57.8.616 12883070 PMC1732540

[B32] Skapinakis P Lewis G Araya R Jones K Williams G Mental health inequalities in Wales, UK: multi-level investigation of the effects of area deprivation Br J Psychiatry 2005 186 417 422 10.1192/bjp.186.5.417 15863747

[B33] De Silva M McKenzie K Harpham T Huttly S Social capital and mental illness: a systematic review J Epidemiol Community Health 2005 59 619 627 10.1136/jech.2004.029678 16020636 PMC1733100

[B34] Muntaner C Invited commentary: social mechanisms, race, and social epidemiology Am J Epidemiol 1999 150 2 121 126 10.1093/oxfordjournals.aje.a009970 10412956

[B35] Krieger N Theories for social epidemiology in the 21st century: an ecosocial perspective Int J Epidemiol 2001 30 668 677 10.1093/ije/30.4.668 11511581

[B36] O'Campo P Invited commentary: advancing theory and methods for multilevel models of residential neighbourhoods and health Am J Epidemiol 2003 157 9 13 10.1093/aje/kwf171 12505885

[B37] Carpiano R Daley D A guide and glossary on postpositivist theory building for population health J Epidemiol Community Health 2006 60 564 570 10.1136/jech.2004.031534 16790824 PMC2566228

[B38] Raphael D Social determinants of health: present status, unanswered questions and future directions Int J Health Serv 2006 36 4 651 677 10.2190/3MW4-1EK3-DGRQ-2CRF 17175840

[B39] Kaplan G What's wrong with social epidemiology, and how can we make it better Epidemiol Rev 2004 26 124 135 10.1093/epirev/mxh010 15234953

[B40] Curtis S Rees-Jones I Is there a place for geograpy in the analysis of health inequality? Sociol Health Illn 1998 20 645 672 10.1111/1467-9566.00123

[B41] Shankardass K Dunn J O'Campo P, Dunn J How Goes the Neighbourhood? Rethinking Neighbourhoods and Health Research in Social Epidemiology Rethinking Social Epidemiology: Towards a Science of Change 2012 Dordrecht, Netherlands: Springer Science + Business Media B.V 137 156

[B42] Miles M Huberman A Qualitative Data Analysis: An Expanded Sourcebook 1994 Thousand Oakes, CA: Sage Publications

[B43] Glaser B Strauss A The Discovery of Grounded Theory: Strategies for Qualitative Research 1967 Chicago: Aldine

[B44] Charmaz K Constructing Grounded Theory 2006 London: Sage Publications

[B45] Saldana J The Coding Manual for Qualitative Researchers 2009 Los Angeles: Sage Publications

[B46] Glaser B Theoretical Sensitivity: Advances in the methodology of grounded theory 1978 Mill Valley: Sociological Press

[B47] Kawachi I Subramanian S Kim D Kawachi I, Subramanian S, Kim D Social Capital and Health: A Decade of Progress and Beyond Social Capital and Health 2008 New York, NY, USA: Springer Science + Business Media 1 26

[B48] Carpiano R Toward a neighborhood resource-based theory of social capital for health: Can Bourdieu and sociology help Soc Sci Med 2006 62 1 165 175 10.1016/j.socscimed.2005.05.020 15992978

[B49] Carpiano R Kawachi I, Subramanian S, Kim D Actual and Potential Neighbourhood Resources for Health: What can Bourdieu offer for understanding Social Capital and Health 2008 New York: Springer

[B50] Putnam R The prosperous community: social capital and public life American Prospect 1993 13 35 42

[B51] Putnam R Bowling alone: America's declining social capital J Democracy 1995 6 65 78

[B52] Putnam R Bowling Alone: The Collapse and Revival of American Community 2000 New York: Simon and Schuster

[B53] Bourdieu P Richardson J The forms of capital Handbook of Theory and Research for the Sociology of Education 1986 New York: Greenwood

[B54] Bourdieu P Wacquant L An Invitation to Reflexive Sociology 1992 Chicago: University of Chicago Press

[B55] Hutchison P Abrams D Christian J Abrams D, Christian J, Gordon D The Social Psychology of Exclusion Multidisciplinary Handbook of Social Exclusion Research 2007 Chichester: John Wiley & Sons Ltd

[B56] Morrell C Warner R Slade P Dixon S Walters S Paley G Brugha T Psychological interventions for postnatal depression: cluster randomised trial and economic evaluation. The PoNDER trial Health Technol Assess 2009 13 30 10.3310/hta13300 19555590

[B57] Dennis C Hodnett E Kenton L Weston J Zupancic J Stewart D Kiss A Effect of peer support on prevention of postnatal depression among high risk women: multisite randomised controlled trial Br Med J 2009 338 a3064 10.1136/bmj.a3064 19147637 PMC2628301

[B58] Roman L Gardiner J Lindsay J Moore J Luo Z Baer L Goddeeris J Shoemaker A Barton L Fitzgerald H Alleviating perinatal depressive symptoms and stress: a nurse-community health worker randomized trial Arch Womens Ment Health 2009 12 6 379 391 10.1007/s00737-009-0083-4 19551471

[B59] Melhuish E Belsky J Leyland A Barnes J Effects of fully-established Sure Start Local Programmes on 3-year-old children and their families living in England: a quasi-experimental observational study The Lancet 2008 372 9650 1641 1647 10.1016/S0140-6736(08)61687-6 18994661

[B60] Kramer M Hogue C Is segregation bad for your health Epidemiol Rev 2009 31 178 194 10.1093/epirev/mxp001 19465747 PMC4362512

[B61] Acevedo-Garcia D Lochner K Kawachi I, Berkman L Residential Segregation and Health Neighborhoods and Health 2003 Oxford, UK: Oxford University Press

[B62] Berry J Poortinga Y Segall M Dasen P Cross-Cultural Psychology: Research and applications 2002 2 Cambridge: Cambridge University Press

[B63] Van de Vijver F Van Hemert D Poortinga Y Van de Vijver F, Van Hemert D, Poortinga Y Conceptual Issues in Multilevel Models Multilevel Analysis of Individuals and Cultures 2008 New York: Lawrence Erlbaum Associates

[B64] Ritzer G Modern Sociological Theory 2008 7 New York: McGraw-Hill

[B65] Beck CT The lived experience of postpartum depression: a phenomenological study Nurs Res 1992 41 3 166 170 1584660

[B66] Beck CT Teetering on the edge: a substantive theory of postpartum depression Nurs Res 1993 42 1 42 48 8424067

[B67] Beck CT Postnatal depression: a metasynthesis Qual Health Res 2002 12 4 453 472 10.1177/104973202129120016 11939248

[B68] Sayer A Realism and Social Science 2000 London: Sage Publications

[B69] Kitto S Chesters J Grbich C Quality in qualitative research. Criteria for authors and assessors in the submission and assessment of qualitative research articles for the Medical Journal of Australia Med J Aust 2008 188 4 243 246 18279135 10.5694/j.1326-5377.2008.tb01595.x

[B70] Greenhalgh T Taylor R How to read a paper: papers that go beyond numbers (qualitative research) Br Med J 1997 315 7110 740 743 10.1136/bmj.315.7110.740 9314762 PMC2127518

[B71] Miyata H Kai I Reconsidering evaluation criteria for scientific adequacy in health care research: an integrative framework of quantitative and qualitative criteria Int J Qual Methods 2009 8 1 64 16813064

[B72] Cohen D Crabtree B Evaluative criteria for qualitative research in health care: Controversies and recommendations Ann Fam Med 2008 6 4 331 10.1370/afm.818 18626033 PMC2478498

[B73] Pawson R Evidence-Based Policy: A Realist Perspective 2006 London: Sage Publications

[B74] O'Campo P Kirst M Schaefer-McDaniel N Firestone M Scott A McShane K Community-based services for homeless adults experiencing concurrent mental health and substance use disorders: a realist approach to synthesizing evidence J Urban Health 2009 86 6 965 989 10.1007/s11524-009-9392-1 19760155 PMC2791817

[B75] Danermark B Ekstrom M Jakobsen L Karlsson J Explaining Society: Critical realism in the social sciences 2002 London: Routledge

[B76] McGuire W Beyond EBM: new directions for evidence-based public health Perspect Biol Med 2005 48 4 557 569 10.1353/pbm.2005.0081 16227667

[B77] Mingers J A critique of statistical modelling in management science from a critical realist perspective: its role within multimethodology J Operations Res Soc 2006 57 202 219

[B78] Haig B An abductive theory of scientific method Psychol Methods 2005 10 4 371 388 16392993 10.1037/1082-989X.10.4.371

[B79] Koenig G Realistic evaluation and case studies Evaluation 2009 15 1 9 30 10.1177/1356389008097869

[B80] O'Campo P Salmon C Burke J Neighbourhoods and mental well-being: What are the pathways Health Place 2009 15 56 68 10.1016/j.healthplace.2008.02.004 18420446

[B81] Kane M Trochim W Concept Mapping for Planning and Evaluation 2007 Thousand Oakes, CA: Sage Publications

[B82] Eastwood J Jalaludin B Kemp B Phung H Realist identification of group-level latent variables for perinatal social epidemiology theory building Int J Health Serv 2014 In Press 10.2190/HS.44.3.a 25618983

[B83] Eastwood J Jalaludin B Kemp L Phung H Barnett B Immigrant maternal depression and social networks. A multilevel Bayesian spatial logistic regression in South Western Sydney, Australia Spat Spatiotemporal Epidemiol 2013 6 49 58 23973180 10.1016/j.sste.2013.04.003

